# Closed-Loop Deep Brain Stimulation for Refractory Chronic Pain

**DOI:** 10.3389/fncom.2018.00018

**Published:** 2018-03-26

**Authors:** Prasad Shirvalkar, Tess L. Veuthey, Heather E. Dawes, Edward F. Chang

**Affiliations:** ^1^Pain Management Division, Departments of Neurology and Anesthesiology, University of California, San Francisco, San Francisco, CA, United States; ^2^Department of Neurological Surgery, University of California, San Francisco, San Francisco, CA, United States; ^3^Neuroscience Graduate Program, University of California, San Francisco, San Francisco, CA, United States

**Keywords:** closed-loop stimulation, deep brain stimulation, neuropathic pain, chronic pain, control theory, somatosensory, affective, cognitive

## Abstract

Pain is a subjective experience that alerts an individual to actual or potential tissue damage. Through mechanisms that are still unclear, normal physiological pain can lose its adaptive value and evolve into pathological chronic neuropathic pain. Chronic pain is a multifaceted experience that can be understood in terms of somatosensory, affective, and cognitive dimensions, each with associated symptoms and neural signals. While there have been many attempts to treat chronic pain, in this article we will argue that feedback-controlled ‘closed-loop’ deep brain stimulation (DBS) offers an urgent and promising route for treatment. Contemporary DBS trials for chronic pain use “open-loop” approaches in which tonic stimulation is delivered with fixed parameters to a single brain region. The impact of key variables such as the target brain region and the stimulation waveform is unclear, and long-term efficacy has mixed results. We hypothesize that chronic pain is due to abnormal synchronization between brain networks encoding the somatosensory, affective and cognitive dimensions of pain, and that multisite, closed-loop DBS provides an intuitive mechanism for disrupting that synchrony. By (1) identifying biomarkers of the subjective pain experience and (2) integrating these signals into a state-space representation of pain, we can create a predictive model of each patient's pain experience. Then, by establishing how stimulation in different brain regions influences individual neural signals, we can design real-time, closed-loop therapies tailored to each patient. While chronic pain is a complex disorder that has eluded modern therapies, rich historical data and state-of-the-art technology can now be used to develop a promising treatment.

## Introduction

**Figure 1:**
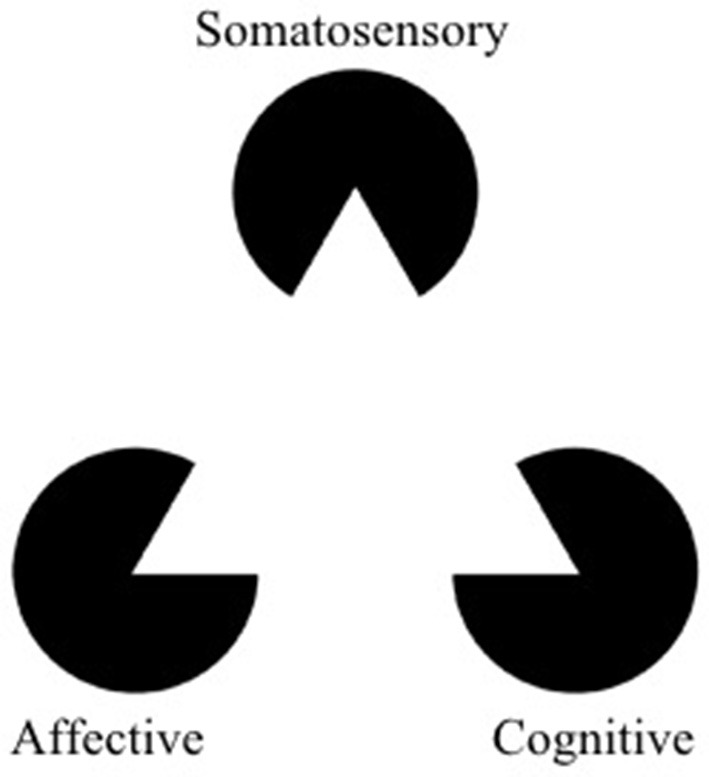
The Kanizsa triangle can be used to represent a multidimensional framework for pain. Pain is an underlying state made apparent by three types of observable symptoms (somatosensory, affective, and cognitive). Therapies which selectively address a single facet of pain risk misinterpreting aspects of symptoms (the “shape” of the symptoms) outside of the context of the larger pathology. The optimal way to “break” the pain state might lie in modulation (or “re-orienting”) the facets of pain rather than trying to suppress them (adapted from https://commons.wikimedia.org/wiki/File:Kanizsa_triangle.svg; Accessed on March 14, 2018).

Chronic pain is a major healthcare problem, and estimates by the CDC suggest that it affects more people in the US than heart disease, diabetes and cancer combined (CDC, 2016). Central neuropathic pain, defined by the International Association for the Study of Pain as pain originating from a lesion of the brain or spinal cord, is often refractory to treatments (IASP)^1^. Common pharmacological therapies have marginal analgesic benefit, and so far, modern neuromodulation therapies such as spinal cord or deep brain stimulation have had limited efficacy over time. Currently, these therapies offer a one-size-fits-all approach that is not optimized for individual neural signatures of pain. However, we believe that central pain syndromes are particularly good candidate conditions for personalized medicine. Each patient's pain is a multifaceted experience that can be understood in terms of somatosensory, affective, and cognitive dimensions, each correlated with activity in different brain regions (Melzack and Casey, 1968; Melzack, 1999; Figure 1). We hypothesize that enduring analgesia will be best achieved by identifying patients' unique neurophysiological biomarkers of pain perception across multiple brain regions and providing tailored, feedback-controlled deep brain stimulation across those target regions. Importantly, we acknowledge that we seek not to abolish all pain perception *per se*, as pain may serve an adaptive role to averting tissue injury. In this article, we outline prior approaches to DBS for chronic pain, an approach to identifying neural biomarkers of pain, and propose strategies to develop a framework for closed-loop DBS based on control theory and state-space paradigms.

## A brief history of DBS for pain

Chronic pain has been conceptualized as a multidimensional process for many decades. Opioids, one of the most common therapies for chronic pain, incidentally provide relief for somatosensory, affective, and cognitive aspects of pain and target top down modulation of pain sensation (Zubieta et al., 2001; Villemure and Bushnell, 2002; Ossipov et al., 2010). However, most neuromodulatory therapies such as transcranial magnetic stimulation (TMS) and DBS still focus on a single facet of pain, originally targeting either somatosensory networks or more recently targeting affective regions. These therapies and their outcomes provide insight into the potential and limitations of addressing centralized pain syndromes as a single-modality pathology.

### DBS for somatosensory pain symptoms

Early efforts at targeting DBS for pain focused on modulating signals in somatosensory networks. Initial inspiration to target these brain regions was inspired by Dejérine and Roussy's descriptions of post-stroke pain syndrome in patients with thalamic infarcts involving the spinothalamic pathway (Dejerine and Roussy, 1906). In an attempt to silence aberrant activity in somatosensory pathways, patients underwent ablations of various segments along the spinothalamic tract and the dorsal thalamus (Wycis and Spiegel, 1949; Tasker, 1990). Eventually, direct electrical stimulation of the dorsal column (Shealy, 1969), internal capsule (Adams et al., 1974) and sensory thalamus (Hosobuchi et al., 1973) provided a reversible alternative to ablation.

Based on results from intraoperative microstimulation in humans, several groups designed studies to disrupt neural signals of different nodes in the somatosensory/nociceptive network. Since 1969, small case series targeting DBS to the ventral (or caudal) thalamus (vT), internal capsule, and periventricular/periaqueductal gray (PVG/PAG) were conducted with efficacy rates ranging from 23 to 59% (Hosobuchi et al., 1973; Adams et al., 1974; Levy et al., 2010). To extend these case series, Medtronic conducted two large, multicenter, randomized controlled trials in the early 1990s for a heterogeneous group of chronic pain conditions (Coffey, 2001). All patients were implanted with bilateral electrodes targeted the vT and PAG. These trials established the primary endpoint still used by most modern chronic pain trials: >50% reduction of the pain visual analog score (VAS) at 1 year. However, they were aborted in the 1990s, largely due to poor enrollment and participant attrition. Around the same time, the FDA granted Medtronic approval of DBS for Parkinson's Disease (PD) and essential tremor and Medtronic never sought market approval for pain indications. Common criticisms of Medtronic's DBS trials for chronic pain include (1) poor patient selection due to wide heterogeneity of pain etiologies (i.e., nociceptive pain, neuropathic pain, thalamic pain, visceral pain, brachial plexus avulsion, unspecified etc.), (2) a minority of purely neuropathic pain syndromes (~30%) and (3) lack of appropriate patient follow up. This study used fixed, tonic stimulation parameters ranging from 100 to 130 Hz which were manually optimized at the start of the study for each patient. It remained unclear exactly how electrical stimulation affected targeted regions, but long-term pain relief waned, likely due to adaptation of the nervous system to continuous stimulation and the development of tolerance. Despite this lack of mechanistic clarity, DBS became a compelling experimental therapy because it is still preferable to permanent ablation or resection of brain tissue which has low analgesic efficacy.

Early attempts to stimulate the somatosensory cortex directly failed to provide pain relief (Hosomi et al., 2015). Instead, stimulation of the adjacent motor cortex with arrays of electrodes has been successfully used to treat pain syndromes such as pelvic pain (Louppe et al., 2013), trigeminal neuralgia (Brown and Pilitsis, 2005) and phantom limb pain (Lefaucheur et al., 2009), presumably by providing feedback inhibition of S1 inputs (Hosomi et al., 2015). Efficacy rates of motor cortex stimulation range from 40 to 60% but significant long-term studies are lacking.

### DBS for affective pain symptoms

Based on animal studies implicating limbic system structures in emotional experience and expression (Papez, 1937; Nauta, 1958), early brain surgery for chronic pain involved anterior cingulotomy to alleviate pain. Case studies of these patients described individuals with intact somatosensation, but who seemed to lack “emotional tension” (Whitty et al., 1952; Ballantine et al., 1967) and lacked “emotional reactivity” to pain stimuli (Foltz and White, 1962) without being emotionally blunted.

The earliest reports of DBS induced analgesia were actually serendipitous findings from stimulation of septal nuclei in patients with psychiatric disorders in the 1950's (Levy et al., 2010). These findings were not followed up until the 1960's, when Lewin and Whitty performed intraoperative stimulation of the cingulate cortex which produced transient analgesia.

Modulating the affective component of pain reflects a paradigm shift for DBS in the twenty-first century. Recent studies measuring cerebral blood flow with positron emission tomography (PET) or functional magnetic resonance imaging (fMRI) have specifically identified the dorsal anterior cingulate (dACC), insula, and dorsolateral prefrontal cortex (DLPFC) as key substrates underlying subjective pain experience (Coghill et al., 2003; Wager et al., 2013) of which the ACC may be specific to the affective component of pain (Rainville et al., 1997). Animal studies have further corroborated this evidence by demonstrating a causal role for ACC neurons in mediating the “aversiveness” of nociceptive stimuli. Fields and colleagues demonstrated that destructive lesions of the rostral ACC reduce learned conditioned pain preference in a rat pain assay (Johansen et al., 2001). Injecting an excitatory amino acid into the ACC, even in the absence of a noxious pain stimulus, actually increases conditioned place preference, suggesting that the ACC is both necessary and sufficient for learning the “unpleasantness” associated with pain stimuli (Johansen and Fields, 2004).

Two cases of ACC stimulation for spinal cord injury have shown therapeutic promise (Spooner et al., 2007), and another recent study demonstrated that stimulation of the anterior midcingulate cortex produced an attitude of resilience and “will to persevere (Parvizi et al., 2013).” The first human clinical trial using open-loop DBS in ACC for chronic pain showed a significant decrease in pain ratings (Visual Analog Score) at 1 year with enduring relief at a 2 year time point (Boccard et al., 2015, 2017). A recent attempt to modulate the affective dimension of pain with DBS targeting the ventral striatum/anterior limb of the internal capsule for post stroke pain did not show improvement in pain scores, but did enhance measures of mood further implicating basal forebrain regions in distributed pain circuits (Lempka et al., 2017).

### Limitations to current approaches

Current clinical paradigms for DBS are all “open-loop” systems, in which tonic stimulation is continuously applied to a single brain region. Constraints on electrode location and stimulation parameters limit the efficacy of open-loop DBS.

#### Anatomical limitations

By restricting stimulation to one brain region, traditional DBS fails to account for the fact that the hallmark of pain is not based on strong signals in any *single* one of the three components of pain (somatosensory, affective, and cognitive), but a confluence of signals in all three (Figure 1). We hypothesize that chronic pain is due to abnormal synchronization between brain networks encoding these three dimensions of pain. Consequently, effective pain relief is unlikely to be achieved by blunting a single component; instead, it will be more effective to decouple and modulate each of them through multisite stimulation. Below, we propose the following candidate brain regions as appropriate targets to test our hypothesis: primary somatosensory cortex (S1, somatosensory), dorsal anterior cingulate cortex (dACC, affective), and orbitofrontal cortex (OFC, affective and cognitive) (**Figure 3**).

#### Stimulation limitations

By restricting stimulation to fixed parameters, an open-loop strategy cannot take into account the fact that pain for a single patient is dynamic and comes in many forms. While some instances of pain are evoked by sensory stimuli, spontaneous and constant pain states are also influenced by mood and attention (Villemure and Bushnell, 2002; Bushnell et al., 2013). Based on personal pain symptoms, abnormal somatosensory signals will need to be modulated to different degrees than affective and cognitive signals in a time varying manner. Currently, stimulation parameters are optimized through trial-and-error by a healthcare provider by systematically changing variables such as pulse width, frequency and amplitude to find the settings that best provide a desired effect. Changes are made on the timescale of patient visits. Ideally, adaptive stimulation would change in real-time to match the dynamic changes in a patient's pain state.

#### Temporal limitations

Tonic, open-loop stimulation also does not account for the dynamic nature of pain or adaptation of the brain over time. Loss of therapy over months to years has been attributed to changing impedance of electrodes and development of scar tissue around contact sites, though more recent evidence implicates adaptation of neural activity to tonic stimulation (Romanelli et al., 2004). A feedback driven stimulation paradigm would ideally account for such adaptation and adjust the contact site or parameters of stimulation appropriately. Closed-loop DBS provides flexible solutions to limitations of open-loop approaches. Below, we describe a theoretical framework for design of a feedback-controlled (closed-loop) DBS system to address the multiple dimensions of chronic pain using state-space control theory.

## Applying control theory to DBS for pain

Pain can be studied, understood, and treated through different levels of abstraction. Prescribing opioids inherently addresses pain as a chemical process. Here we will address pain as a network process. Through this lens we will analogize pain to a dysfunctional signal within an electrical network, which itself is limited to a few components within the central nervous system. In this analogy, managing pain can be addressed as a control systems problem, in which the brain is the component we are trying to regulate, and the DBS device is the control box. The availability of different control systems, particularly open-loop vs. closed-loop devices, leads to different goals and approaches. However, no artificial system will be a full substitute for a healthy human pain system, which relies on access to widespread brain regions to provide pain control that is influenced by mood, social context, physical modality, emotional valence, attention and temporal structure. We suggest that both open-loop and closed-loop strategies should set realistic goals, such as identifying and preventing both constant and spontaneous pain states and/or giving patients more control over their pain treatment.

### Mapping DBS onto a control framework

**Figure 2:**
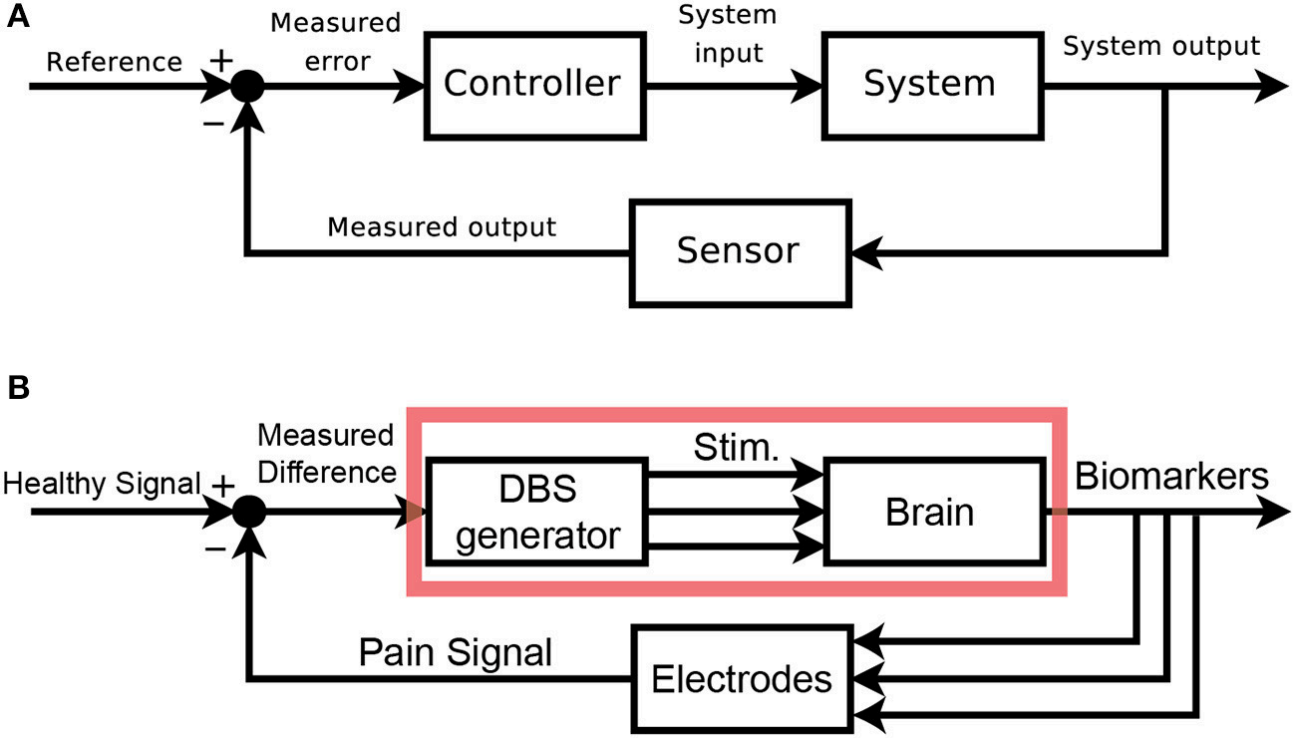
Block Diagram schematics of closed-loop control systems. **(A)** Classical block diagram of a single-input, single-output negative feedback control system, where the measured output of the system is compared to a reference signal via a closed-loop, to modify the system output and minimize error [adapted from (Orzetto)]. **(B)** Example block diagram of a multi-input, multi-output closed-loop DBS system where a pain signal derived from biomarkers is compared to a reference signal via a feedback loop. Multi-regional stimulation is triggered to bring the system closer into the reference state. The red box highlights elements of an open-loop paradigm. ^a^Available online at: https://upload.wikimedia.org/wikipedia/commons/2/24/Feedback_loop_with_descriptions.svg (Accessed Nov 30, 2017).

We would like to clearly map out the analogy between classic control schemas and pain control through external devices. Figure 2A shows the classic layout of a feedback-driven control system, and Figure 2B shows how different components of DBS as a medical intervention map onto each role. The system in question is the brain itself, specifically the pain-related regions with pathological pain signals. The system output is an observable biomarker which we hypothesize as giving an accurate, relevant, and temporally appropriate view into the patient's pain state. The sensor is any implanted recording electrodes (e.g., microwire arrays, ECoG grids, EEG leads), which records neural signals. The reference signal is the desired version (pain-free) of the neural signal. A closed-loop device would compare the sensed neural signals to the reference signal (measured difference) and trigger the DBS device (controller) to appropriate corrective stimulation, with the assumption that stimulation can control the relevant internal state of the patient.

An **open-loop** system would be limited to the components in the red box (Figure 2B). Since there is no sensor, the output of this system is the patient's self-report of pain. The healthcare provider compares this self-report to a reference, pain-free state and can adjust DBS stimulation parameters as needed. The timescale of updates is clinic visits, and there is no view into underlying neural signals related to pain. A **closed-loop** system (minimally defined as any system with in which stimulation is based on a sensor readout) gives access to neural signals that are interpreted as real-time proxies for the patient's internal pain state. This readout is fed back and compared to a reference neural signal. Based on the difference between these signals, a controller makes responsive, real-time adjustments to stimulation parameters. It is the hope that closed-loop paradigms will improve outcomes and reduce side-effects compared to open-loop paradigms.

#### State space models

State-space representations are used in control engineering to model systems with multiple inputs, multiple outputs and latent state variables which can be used to represent dynamic sequences of brain states (Smith and Brown, 2003; Hsieh and Shanechi, 2016). Neural state-space representations can consist of a number of time dependent input variables, such as firing rates from neurons or local field potentials (LFP) power time series from multiple recording channels. If the number of variables (i.e., neurons or electrode contacts) is very large, it is useful to first reduce the dimensionality of the data to a set of orthogonal dimensions that describes the phenomena of interest with fewer variables (Cunningham and Yu, 2014). This dimensionality reduction is commonly done with tools such as principal components or factor analysis which can help to identify *latent* variables that define a new coordinate system. Temporal evolution of the neural signal through this coordinate system can be interpreted as “neural trajectories.”

Recently, state-space representations have been used to understand the evolution of neural signals from motor cortex during reaching tasks (Churchland et al., 2006; Shenoy et al., 2013). The relationships between external triggers (visual reach target onset, go cue), internal state (movement preparation), conscious experience (anticipation), and behavior (movement onset) are intuitive for motor processes, and we argue that applying state-space analysis to pain dynamics may be similarly useful. While dynamical systems analysis of movement has so far mostly relied on single-neuron signals, there are also ample reports of using LFP from motor cortex to decode movements and screen cursor location (Flint et al., 2012; Orsborn et al., 2013; So et al., 2014; Stavisky et al., 2015). Because shifts in pain state are slow, multiregional neural phenomena, we predict that LFP changes across multiple brain regions will provide a temporally appropriate neural report of pain state fluctuations. Multivariate data such as LFPs from multiple brain regions can be represented in a “state-space” for pain (**Figure 4**). These are particularly appropriate for analyzing multidimensional phenomena like dimensions of pain. In the next section, we will outline the specific nature of the neural signals which can be interpreted as biomarkers of internal pain states.

### Local field potentials are the most tractable signal for identifying biomarkers for closed-loop DBS

Candidate neurophysiological biomarkers for chronic pain can be derived from three types of signal: single action potentials, LFP within specific frequency bands, and blood oxygen level dependent (BOLD) signals.

Single action potentials are the neural signal with the highest temporal and spatial resolution. However, action potentials collected from chronically implanted tungsten or silicone probes are unstable due to probe drift and sensitivity to behavioral context (i.e. sensory stimulation, arousal state, etc.). Single action potentials from S1 and ACC were used in a rodent model of acute thermal pain to decode a pain state defined through use of a Hidden Markov Model (Chen et al., 2017). In this experiment, signals from the population of single neurons used to computed baseline and pain states were not stable over even a few trials, making the chronic computation of a pain state untenable. Assuming that recorded action potentials from human patients would experience similar instability, chronic biomarkers based on these signals are not tractable. A potential work-around would be to calculate biomarkers based on dynamics from population neural firing combined with high frequency local field potential, a promising strategy used in human brain-machine interfaces (Pandarinath et al., 2017).

Local field potentials represent aggregate population subthreshold activity among a spatially localized population of neurons (Buzsáki et al., 2012). While the term LFP usually refers to signals captured by implanted depth electrodes or cortical electrodes, LFP is thought to reflect brain oscillations similar to those captured by intracranial electroencephalography (iEEG) and magnetoencephalography (MEG). Previous attempts at decoding subjective pain intensity with resting state EEG (Schulz et al., 2012) or MEG (Kuo et al., 2017) have used time-frequency representations of brain oscillations with high accuracy, supporting the feasibility of using LFP to define a pain state. Additionally, LFP signals are (1) easier to record than spikes or evoked potentials over single trials (2) often highly reproducible within an individual and (3) can be examined by well-developed signal analysis tools (Bokil et al., 2010). Previous studies of Parkinson's disease have successfully used LFP from depth electrodes and cortical strips to define biomarkers for tremor and dyskinesia over many days/months, providing support for the stability and longevity of this signal type (Hemptinne et al., 2015). In a closed-loop DBS trial for chronic pain, the healthcare team could record from multiple brain regions simultaneously to track changes in the multiple parallel dimensions of pain: somatosensory (S1, vT, insula), affective (ACC, medial thalamus, and striatum) and cognitive (PFC, OFC, insula).

Finally, several studies have used blood-oxygen-level dependent contrast imaging (BOLD signals) to detect and define pain states in fMRI research (Wager et al., 2013; Lieberman and Eisenberger, 2015; Reddan and Wager, 2017). BOLD signal can reflect brain activity at a spatial resolution under 1 mm and at a temporal resolution of a few seconds (Goense et al., 2016), providing excellent whole-brain localization and temporal tracking of neural activity correlating with pain states. Unfortunately, these signals are not available in the ambulatory setting, prohibiting their use in chronic patient therapy. Also, current closed-loop DBS probes are not MRI compatible, and it is unclear whether these probes would cause signal artifacts once they are implanted. However, asking patients to complete a pre-implantation fMRI study to capture neural signals correlated with spontaneous and evoked pain would be extremely useful to direct patient-tailored anatomic targeting of the probe implant. Ideally, the healthcare team would capture simultaneous fMRI and EEG signals, which could also inform the initial search for LFP-based biomarkers (assuming that LFP signals provide a local view of neural signals more grossly captured in EEG; Huster et al., 2012).

## Computing a pain state from regional biomarkers

Pain is a multi-faceted process that can be broken down into somatosensory, affective, and cognitive components (Melzack and Casey, 1968). Each component can be associated with distinct symptoms and brain regions. Importantly, information processing for each component is not fully segregated, but instead involves activity in overlapping neural pathways. Currently, constellations of somatosensory, affective, and cognitive signs and symptoms are integrated by healthcare providers to characterize each patient's pain state. For example, two patients with back pain might have different locations and intensities of pain and might also be more or less bothered and distracted by that pain. Ideally, a complete description of a patient's pain state contains all of these components.

**Figure 3:**
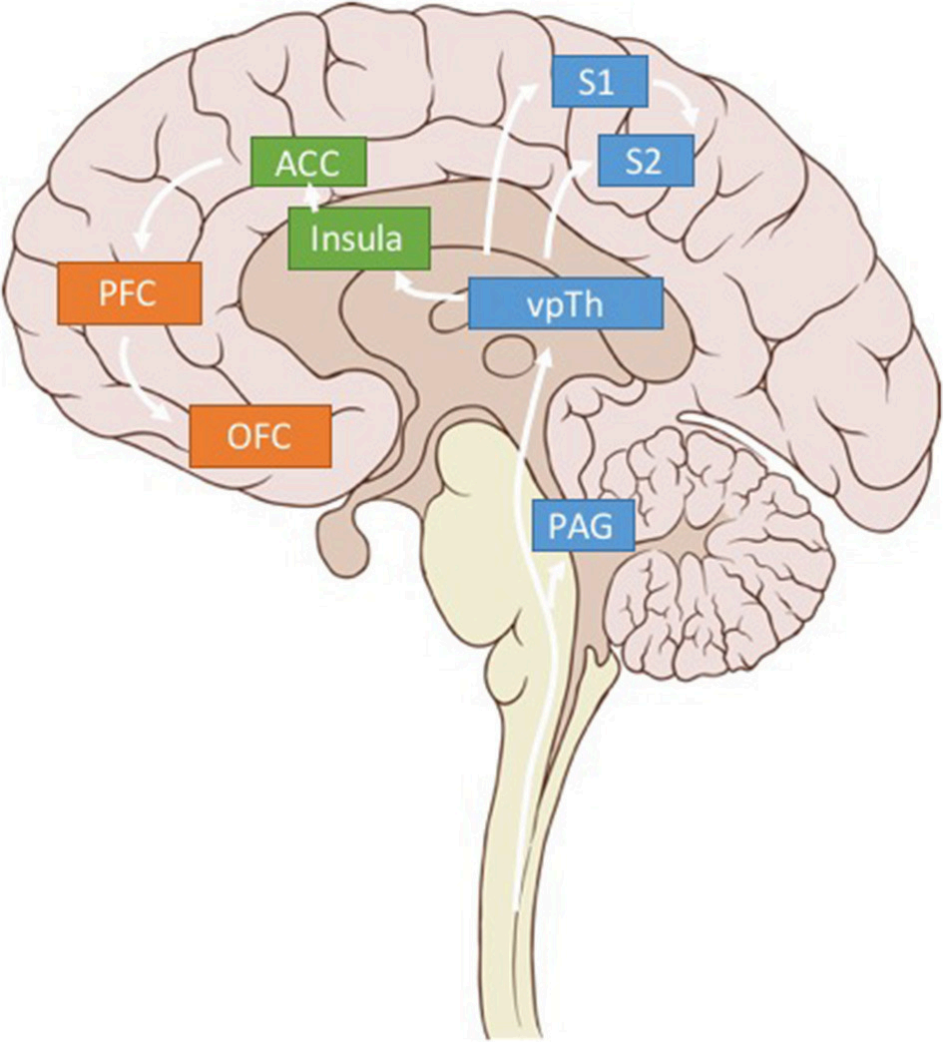
Pain related brain regions. Key brain regions related to somatosensory (blue), affective (green), and cognitive (orange) pain processing. Only regions of interest have been included for clarity.

Similarly, neural recordings from different brain regions could be integrated to provide a multidimensional neural signature of a patient's pain state (Figure 3). Through this neural report, closed-loop brain stimulation becomes a tractable strategy for addressing dynamic pathological brain states. Based on real-time representations of a patient's pain within a neural state space, a closed-loop system can stimulate different brain regions to normalize different components of pain. Such a real-time representation of pain requires accurate and reliable detection of neural biomarkers for somatosensory, affective, and cognitive components of pain. Like patient-reported symptoms of pain, these biomarkers can be thought of as the observable markers of the pain state.

We argue that using LFP signals from three brain regions—S1, dACC, and OFC—could be used to calculate multidimensional, patient-specific pain states (Figure 3). (While we believe these brain regions are critical sites for detecting pain signals there are other valuable regions that have been omitted for clarity in Figure 3). Each patient's biomarkers will need to be determined empirically, but prior literature (elaborated below), suggests high gamma power in S1, high gamma and low alpha power in dACC, and low alpha power in OFC as reasonable starting points.

### Somatosensory signals

The somatosensory-discriminatory component of pain encompasses the intensity, location and duration of a noxious stimulus (“what,” “where,” and “when”). This component of pain has been the most widely studied and is often modeled with transient acute painful stimuli such as electric shock or a phasic thermal or laser pain stimulus lasting a few seconds. As a first step toward decoding chronic pain states, it may be helpful to study decoding of acute pain stimuli, though it is critical to distinguish biomarkers of pain perception from mere pre-perceptual stimulus processing. Human functional imaging data point to a widely distributed neural network that is activated by acute experimental pain perception including the primary and secondary somatosensory cortex (S1 and S2), insula, ACC, PFC and thalamus (Coghill et al., 2003; Apkarian et al., 2005; Wager et al., 2013). However, not all signals can strictly be interpreted to represent somatosensory perception.

A recent study using magnetoencephalography (MEG) to identify neural correlates of cutaneous laser evoked pain in healthy human subjects showed an increase in gamma band amplitude (65–90 Hz) in the contralateral S1 at 200–400 ms post stimulus onset (Gross et al., 2007). This gamma increase was predictive of subjective pain intensity and persisted when controlling for stimulus salience or attentional (cognitive) effects by presenting a stimulus repeatedly (Zhang et al., 2012). Therefore, gamma activity may represent pain perception and not just stimulus processing. However, many of these studies lack non-painful control stimuli, making it possible that gamma activity reflects somatosensation more generally.

Baseline EEG recordings of patients with chronic neuropathic pain show increased theta (4–10 Hz), alpha (12–20 Hz), and beta (20–30 Hz) band power in the insula, frontal cortices, and anterior cingulate (Sarnthein et al., 2006; Stern et al., 2006) which may reflect multiple pain dimensions. There is a further trend toward global slowing with lower peak alpha and theta frequencies in patients with neuropathic or thermal pain (Boord et al., 2008) which is not seen in nociceptive pain (Schmidt et al., 2012). Further, suppression of alpha band oscillations is commonly reported after acute pain stimuli (further discussion below, Ploner et al., 2006). Together these data point to band-limited power changes in S1, insula and thalamus as candidate biomarkers for somatosensory- discriminative pain perception.

Given the pragmatic need to select a single somatosensory region from which to derive pain signals, we suggest recording in S1 rather than vT because cortical regions have higher amplitude signals and may be more reliable over time. While optimal somatosensory biomarkers are best determined empirically for each patient, filtered high gamma power has been a consistent marker in several studies and provides a reasonable starting point as a feedback-control signal for closed-loop DBS. After correlating the relationship of gamma power to patient-reported pain scores, values for gamma power that reliably distinguish high pain states from low pain states can be used to define a high pain-state detection threshold. Then, threshold crossing of real-time gamma power, in combination with other regional biomarkers, can be used to automatically activate analgesic stimulation as needed (see section Pragmatic Considerations for a Closed-Loop DBS Protocol for details).

### Affective signals

The affective dimension involves the “unpleasantness” of a stimulus, and is tied to motivation to rid the pain, changes in mood and anxiety and the degree of suffering (Bushnell et al., 2013). Brain regions underlying affective encoding were identified using positron emission tomography (PET) in subjects undergoing hypnosis to selectively reduce the “unpleasantness” of acutely painful stimuli (Rainville et al., 1997). While individuals still felt similar intensity of pain stimuli under hypnotic suggestion, they were not bothered by these stimuli and they showed reduced activation of the ACC (but not S1) which was linearly related to pain unpleasantness. The role of the rostral ACC in the affective dimension of pain is also corroborated by a recent large meta-analysis of over 10,000 functional MRI datasets (Lieberman and Eisenberger, 2015) and animal studies that support the role of the medial ACC in transition from acute to chronic pain which has a larger affective component (Nevian, 2017).

Tonic pain stimuli lasting longer than 10 min are likely closer to modeling chronic pain states, and engage distinct brain regions from acute pain stimuli (Ploner et al., 2017). EEG recordings in humans point to increased amplitude of gamma band oscillations in the cingulate and medial prefrontal cortex after tonic pain stimuli (Schulz et al., 2015; Li et al., 2016a).

Animal studies also help to identify brain regions and candidate signals that may serve as affective biomarkers of pain perception. In a study recording single spikes from S1 and ACC of rats, a state space model was used to identify neuronal codes underlying acute painful thermal stimuli that produce a paw withdrawal reflex (Chen et al., 2017). One key insight from this study was that population spiking activity from S1 provides better sensitivity for acute pain prediction, while activity from ACC provides better specificity suggesting that a subset of neurons in ACC encode pain information. Simultaneous single neuron recordings in mice in S1, vT, ACC, and mediodorsal thalamus (MD) show temporal and lateralized segregation of encoding of noxious stimuli (Wang et al., 2003). While S1 and vT cells predominantly fired early and contralateral to the pain stimulus, MD and ACC cells had long lasting firing which correlates with the longer time course of pain related anxiety or mood. These data further support the role of the MD thalamus or ACC in affective pain processing. Because cortical signals provide easier surgical access and higher amplitude LFP signals, ACC would be a reasonable initial brain target.

### Cognitive signals

Cognitive aspects of the pain experience involve implementing successful coping strategies, pain anticipation/expectations and behaviors related to attention and distraction (Bushnell et al., 2013). Increased attention to a painful stimulus will increase the perceived intensity of pain without altering its unpleasantness; distraction from pain can be analgesic. Further, pain itself often interferes with attentional processes, making causal inference of the role of attention difficult. Cognitive strategies that reduce pain perception such as distraction increase the amplitude of EEG activity in the DLPFC, orbitofrontal cortex (OFC) and caudal ACC shortly after a pain stimulus (Moont et al., 2012). Modulation of the alpha rhythm is widely associated with the cognitive component of pain. Intracranial recordings in epilepsy patients suggests that increased attention toward a painful stimulus is correlated with alpha and beta band activity in the medial PFC and parasylvian regions that exert a causal influence over S1; this relationship is the opposite with distraction (Liu et al., 2011). Similar alpha coherence between PFC and S1 is seen in ECoGs during pain anticipation (Ohara et al., 2006). Further, the amplitude of frontocentral alpha correlates with subjective expectation of pain relief induced by placebo (Li et al., 2016b). These observations support the role of perisylvian regions such as PFC and OFC, and alpha band oscillations in the cognitive dimension of pain.

Oscillations before the onset of pain can shape the experience of pain and may serve as a context dependent biomarker of cognitive control over pain. Two recent studies show that the amplitude of pre-stimulus alpha oscillations (12–20 Hz) over somatosensory cortex is inversely correlated with pain perception (Babiloni et al., 2006; Tu et al., 2016). However, multiple other studies report changes in alpha power of the PFC with attention and perception of non-painful stimuli, confounding general interpretation of this effect. Functional imaging and EEG studies further point to functional connectivity between the PFC, anterior insula and temporoparietal junction that form a “salience network” that underlies cognitive control over pain (Kucyi and Davis, 2015).

Based on the available literature, OFC would be a reasonable initial target to identify putative pain biomarkers of the cognitive-evaluative dimension.

### Multidimensional biomarkers for chronic pain

By simultaneously recording intracranial LFPs in multiple brain regions, it may be possible to identify biomarkers for unique pain states (spontaneous pain flare, evoked pain, and baseline pain) that are more sensitive and specific than any single brain region can provide. Further, frequency band-limited activity between these brain regions is interpreted to reflect the flow of information (Ohara et al., 2006; Colgin et al., 2009; Ploner et al., 2017), more accurate prediction of pain states may result from calculating phase coherence or amplitude co-modulation between each region's signal. Recent evidence suggests phase or amplitude relationships *between* different frequency oscillations *within* a brain region may also be informative about information flow (Sarnthein et al., 2006; Shirvalkar et al., 2010; Tort et al., 2010) as in a model of closed-loop DBS for Parkinson's Disease (Hemptinne et al., 2015).

We argue that using LFP signals from three brain regions—S1, dACC, and OFC—could be used to calculate multidimensional, patient-specific pain states. Patients' pain states endogenously fluctuate through the day, with higher pain experienced at some time points (e.g., mornings, evenings) and lower pain states expected during periods of rest, sleep or after medication. To identify biomarkers of pain-states, we envision a protocol that involves sampling neural recordings and coincident pain scores during a wide range of naturally fluctuating chronic pain states in the ambulatory setting. Once neural data are collected, they can be transformed to a time-frequency representation to calculate power spectral density. Then, spectral density values within bands of interest (theta, alpha, gamma, etc.) should be used as independent variables to predict pain scores (dependent variables) (see section Pragmatic Considerations for a Closed-Loop DBS Protocol). The most predictive variables or combination thereof would serve as optimal biomarkers from each brain region.

**Figure 4:**
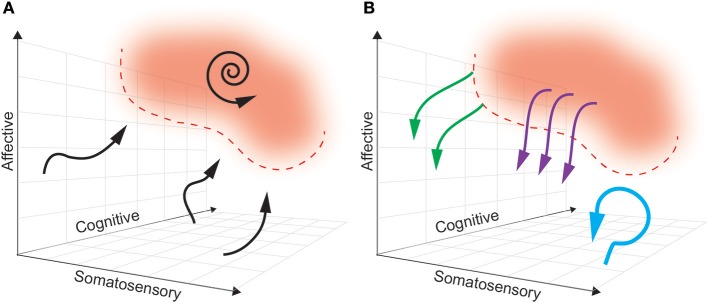
A multidimensional state space framework can be used to characterize pain states, reference states, and goals of DBS paradigms. **(A)** A state space representing neural activity can be defined along the multiple dimensions of pain: somatosensory, affective and cognitive. For simplicity, a pain state is represented as a single red zone in the upper right corner, with defined threshold boundaries (dashed red line). The reference (pain-free) state is any region outside the red zone. The dynamics of neural activity that underlie transition from a pain-free state toward a pain state are shown as neural trajectories (black arrows). During constant baseline pain, there is a self-sustaining neural trajectory confined to the pain state (spiral arrow). **(B)** Different paradigms of DBS accomplish different goals. Tonic, open-loop DBS aims to maintain neural activity in a constant pain-free state (blue arrow). Abortive, patient-triggered or sensor-triggered DBS aims to push neural representations out of the pain state into the reference state (purple arrows). Closed-loop DBS will ideally deflect neural activity well before entering a pain-state (green arrows).

Ideally, a multidimensional biomarker (based in regions relevant to dimensions of pain) will define a pain “landscape” that will distinguish pain states to be avoided from pain-free states that are desired (see Figure 4). In this theoretical framework, the next challenge is characterizing the dynamics of how brain activity in the above regions naturally enters and exits this pain state. As such, the boundaries of a pain state biomarker can be established by setting an appropriate threshold. The ideal goal of a closed-loop DBS paradigm is to prevent the onset of a pain state, rather than simply aborting it once it has commenced. By characterizing the causal consequences of different patterns of brain stimulation, we can determine the optimal stimulation parameters needed to avoid pain states, at multiple points in the landscape. Neural activity is adaptive, however, and this pain landscape may evolve over time making it difficult to define stable boundaries of pain-free states.

### Computing a reference state

We can define a pain-free reference state with the same protocol (section Multidimensional Biomarkers for Chronic Pain) used to define the boundaries of a high pain-state (Figure 4). The role of a reference state is to define the range of biomarker values which in turn will guide selection of a threshold to trigger stimulation. In practice, a “reference” state would reflect any value of the biomarker below a defined threshold for high-pain (i.e., NRS > 7). In this view reference can simply be interpreted to mean “non-high pain state.” Empirical data from chronic human recordings is needed to understand the stability of pain-state detection thresholds. Higher instability would require more frequent re-calculation in order to provide a meaningful contrast between the reference and pain states. Ideally, such a signal would be usefully stable on the order of months, but it may be reasonable to perform automated re-calibration monthly or weekly. Potential lapses in therapeutic stimulation can be recognized by the patient who can trigger recalibration to update the model. This updating will entail definition of new pain-state thresholds and the selection of new stimulation parameters.

Alternatively, a reference state can be interpreted to mean a “low pain-state” where numerical pain scores would be < 3, for example. The possible utility of separately defining such a reference state has been suggested by a computational model for closed-loop control to treat essential tremor in non-human primates (Santaniello et al., 2011). In this model, investigators developed a closed-loop control system that automatically adjusted DBS stimulation amplitude based on the spectral content of simulated LFPs from a cohort of 100 neurons in the Vim thalamus. Optimal DBS output to suppress tremor replaced the tremor-related pathological.

LFP spectrum with LFP patterns similar to those simulated in a “reference” tremor-free state. Similar approaches may help control stimulation amplitude in multiple brain regions based on expected “pain-free” regional LFP spectra.

## Modulating a pain state with different stimulation paradigms

Analogous to how biomarkers are selected to best delimit a pain state, stimulation parameters must be optimized to best control the pain state-space trajectory (Figure 4A). The pre-defined goals for stimulation control are to either abort or avoid pain states. The stimulation goal and parameter selection will depend on the control paradigm: open-loop, patient-triggered, sensor-triggered (on/off), or true closed-loop.

### Open-loop stimulation

In an open-loop paradigm, the goal must be to avoid pain states because there is no sensor available to detect them (which would be necessary to abort them). Therefore, the stimulation parameters must be chosen to maintain the neural state in the pain-free zone (see Figure 4B). We hypothesize that this is best accomplished by consistently de-coupling the neural signals in each pain-related region. For example, leads in S1 and ACC could be alternately pulsed at high gamma frequencies to disrupt the ability of the two regions to develop pathological coherence. If only one stimulation site is available, we favor targeting ACC rather than S1 or OFC, given recent promise in clinical trials (Boccard et al., 2017). Decoupling ACC from other regions might be accomplished by tonically inputting local entrainment signals that would block information flow about inappropriate pain. Similarly, local decoupling has been proposed as a hypothesized target for the treatment of hyperkinetic states in DBS for Parkinson's Disease (Swann et al., 2016).

Once the stimulation is turned on, the goal is indefinite avoidance of a pain state. However, onset of the therapeutic effect may take a few days, as continuous pain states fluctuate on the time course of days, and we expect the pain dynamics to have some “inertia.” In a recent trial of open-loop ACC stimulation for chronic pain (Boccard et al., 2015), there was a wash-in period of many days for any therapeutic effect.

### Patient-triggered stimulation

In a patient-triggered paradigm, the goal is to abort pain states detected by the patient. Effective stimulation must be able to halt pain quickly, making the therapy more suitable to modulation of transient, breakthrough pain. Somatosensory signals are the best candidates for interruption in a single-region stimulation paradigm because we assume they have faster dynamics and often begins “upstream” in the pain triggering process. We theoretically favor targeting ventral thalamus or motor cortex (adjacent to S1) for single-region, patient-controlled gamma-frequency stimulation, based on previous partial success of these therapies (Lima and Fregni, 2008; Keifer et al., 2014). Long-term tolerance to stimulation seen in previous vT trials might be prevented by limiting stimulation to brief, patient-triggered periods. However, because chronic pain can be also triggered by affective and cognitive events, such as stress and rumination, a somatosensory-only detection paradigm leaves patients vulnerable to breakthrough pain. Multi-region stimulation in S1 and ACC (or OFC) would aim to de-couple these regions, but the insidious time course of pain dynamics in ACC and OFC may belie optimal control of breakthrough pain. Prior work with “preventative” devices for epilepsy had a 40% failure rate in preventing seizures, highlighting the limitations of an abortive strategy for neuromodulation (Ben-Menachem et al., 1994). Altogether avoiding entry into pain states requires online tracking of the pain state's ongoing dynamics.

### Sensor-triggered stimulation

Instead of relying on external input, a fixed stimulation protocol can be triggered based on the detected position and/or trajectory of the state within in the neural manifold (Figure 4B). To make this possible, the device must include sensors that can detect relevant biomarkers and an algorithm to decode the pain state with a latency short enough to allow intervention. This is commonly referred to as adaptive DBS (aDBS). As we hypothesize that continuous pain states arise from maladaptive coherence between regions involved in in pain processing, multi-area coherence may be an ideal signal to track the underlying neural state. We propose tracking gamma coherence between S1, ACC, and OFC. Preliminary recordings from pain elicited by somatosensory and cognitive events (i.e., touch, asking the patient to attend to their pain) would allow investigators to determine a threshold of gamma coherence to characterize the pain state. Thereafter, coherence values close to that threshold would trigger de-synchronized stimulation in each region in order to prevent further evolution of inter-regional coherence. Side-effects of S1-OFC stimulation are unknown, however. It is possible that pain dynamics may evolve too rapidly to be interrupted before a noticeable pain threshold is breached, leading to breakthrough pain. Decreasing the threshold for allowable coherence may address this shortfall. Overall, sensor-triggered stimulation is a reasonable staring point in the quest to develop new feedback-controlled paradigms. A promising alternative is to implement a closed-loop paradigm with the possibility to continuously manipulate underlying neural states.

### Closed-loop stimulation

In a truly closed-loop system (as we define it), unique stimulation patterns are delivered based on the real-time predicted course of the pain trajectory. This is distinguished from sensor-triggered stimulation in that in a closed-loop protocol, the same coordinate location in a state space may trigger different stimulation patterns or update stimulation parameters (pulse width, frequency, amplitude) dependent on the history and context of the neural trajectory. For this to be possible, we must create a predictive model of the multidimensional pain state such that the future path of each trajectory can be determined based on history and the current state (Churchland et al., 2006; Mante et al., 2013; Figure 4A). There are several methods for producing such a model, including (1) modeling the state as a three dimensional flow field (Rabinovich et al., 2012; Ashwin et al., 2016), or (2) creating a map outlining the probability of transitioning from any point in the field to every other point (i.e., Hidden Markov Models, Radons et al., 1994).

Based on the assumption that stimulation can control or influence neural trajectories related to pain, the model must additionally contain predictions about the effect of stimulation on the pain trajectory (Figure 4B). Typically, characterizing the input-output (IO) relationship between stimulation and neural state in DBS is a painstaking manual process whereby a clinician systematically varies stimulation parameters (pulse width, amplitude, frequency) and records consequent changes in the neural state (Kumar, 2002; Volkmann et al., 2002). A promising method to automate stimulation parameter optimization involves the use of a “binary noise” modulated stimulation pattern whereby a range of parameters are stochastically sampled and used for stimulation (Yang and Shanechi, 2016). With simultaneous neural recordings, one may use binary noise to define IO dynamics of a closed-loop DBS system more efficiently. However, both of these methods risk producing uninterpretable IO relationships if the timescale of stimulation parameter changes does not match the timescale of state space changes (e.g., long wash-in latency for therapeutic effect).

There are many pragmatic barriers to implementing a truly closed-loop system. First, only few devices with dual sensing and stimulating functions are approved for chronic implant in humans: NeuroPace RNS, while other devices are investigational only: Braingate system and Medtronic Activa PC+S device (Hochberg et al., 2006; Sun and Morrell, 2014; Swann et al., 2018). Because there are no chronic, invasive cortical recordings from candidate stimulation regions in patients with chronic pain, it is unclear whether the hypothesized biomarkers will provide sufficient observability of the internal pain state. Second, while computing multi-dimensional state spaces from neural data is routinely done to visualize offline data, implantable devices have not been optimized to perform these computations online. It is unclear what amount of computation will be viable to perform for continuous pain monitoring. Third, because there have been few long-term successes from small-scale trials of DBS for pain, it is unclear whether chronic pain states will be controllable via stimulation. Resolving this uncertainty will require a chronic, multi-site, sensing and stimulating device that allows for rapid exploration of a large range of stimulation parameters. Finally, one of the hopes for closed-loop stimulation is that it will allow for a reduction of current dosage (relative to continuous stimulation in open-loop paradigms), thereby increasing the device's battery life and reducing the side effects of unnecessary stimulation. However, optimizing stimulation based on battery life will require additional trade-offs, such as deciding on a pain threshold at which stimulation will be initiated, limiting duration of stimulation bouts to the minimum required for pain relief, and potentially sacrificing benefits of long-term stimulation, such as learned desynchronization of pain-related regions.

## Conclusion

### Pragmatic considerations for a closed-loop DBS protocol

Above we provide evidence that spectral power of oscillations within specific frequency bands (e.g., theta, alpha, gamma) shows changes in relevant brain regions that may predict low or high pain states. By recording theta, alpha and gamma oscillations from the LFP signal in S1, ACC and OFC during natural fluctuations in a patient's chronic pain state, we can compute spectral power density during periods of high pain states and so define a neural state space model for predicting chronic pain. For this purpose, the low pain state can be interpreted as a “baseline” or reference state.

To compute a time-frequency representation of the raw LFP signal, we use a variant of the discrete Fourier Transform (DFT). There are multiple methods to implement a DFT- we prefer using the multitaper DFT implemented in the Chronux Toolbox for MATLAB, which reduces broadband bias (Bokil et al., 2010). To adequately sample pain states, we propose to use at-home, patient-triggered recordings be collected. Two data collection schemes can be used as needed (1) 60-s recordings can be scheduled at pre-set time points throughout the day (e.g., 8 a.m., 12 noon, 4 p.m., 8 p.m.) or (2) activated by patients by pressing a button on their DBS programmer. Self-reporting of pain numerical rating scores can be done via an automated text-messaging system. For convenience, patients can be prompted up to 4 times per day to report pain scores and trigger recordings if pre-scheduled recordings are not set. Once a series of pain scores spanning a wide range (at least 5 different values on the numerical rating score) are collected, putative biomarker features can be used (as independent variables) to predict high (>7/10) or low (< 4/10) pain score (dependent variable).

One possible solution to predicting low vs. high pain states is to use multivariate logistic regression using biomarker features as independent variables, and low/high pain state as the dichotomous independent variable to be predicted. For example, if the ACC signal shows increased gamma power, and OFC shows decreased theta power during high pain states compared to baseline in an individual patient, predictive value of ACC gamma and OFC theta would be established through a multivariate logistic regression to predict pain state. A classification table would be used to calculate the probability of false positives and negatives, and overall prediction accuracy. In depth methods for developing multivariate classifiers based on logistic regression have been presented previously (Hastie et al., 2009), as has their personalized application to closed-loop DBS systems based on brain-state (Ezzyat et al., 2017). Using receiver operating characteristic (ROC) curves, one could then calculate optimal threshold values for each biomarker such that real-time crossing of ACC gamma or OFC theta power above/below this threshold would activate stimulation. This scheme represents a sensor-triggered protocol which is a good first-step approximation to building a fully closed-loop system that would adjust stimulation amplitude or other parameters based on ongoing neural activity. Solutions to developing fully-closed loop algorithms and optimizing stimulation based on biomarkers have been suggested by computational studies. Recent models have used LFP spectra (beta and gamma power) as feedback-control signals to provide efficient and selective target stimulation in Parkinson's Disease (Karamintziou et al., 2017) and essential tremor (Santaniello et al., 2011). Using finite element modeling, anatomical models from imaging data can be combined with electrical models to optimize how current is delivered from the DBS electrode (Xiao et al., 2016). Further, stimulation patterns derived from computational evolution models may provide more battery-efficient stimulation protocols that can augment energy savings afforded by closed-loop DBS (Brocker et al., 2017). While explicit models have not been reported for closed-loop control in chronic pain states, future studies will need to incorporate multiregional brain recording and stimulation in relevant areas to provide analgesic closed-loop DBS.

As of the writing of this paper, our group is currently enrolling patients for participation in a feasibility study to develop closed-loop DBS algorithms for chronic neuropathic pain (ClinicalTrials.gov ID# NCT03029884). This trial seeks to enroll 10 patients with refractory neuropathic pain syndromes over 2 years and aims to develop a personalized treatment for multiple pain disorders using the Medtronic Activa PC+S device.

## Discussion

The current article makes several important assumptions to create a simple theoretical framework for implementing closed-loop DBS for chronic pain syndromes. First, to disentangle biomarkers related to the somatosensory, affective, and cognitive components of pain, we impose artificial distinctions between brain regions that underlie each dimension of pain. We do not actually believe that chronic pain can be divided into three segregated, independent components with corresponding brain regions Rather, coalitions of cells in specific brain regions provide overlapping and complementary information about pain states.

Second, we would like to acknowledge that DBS provides an artificial input that may drive neural signals into unnatural regions of the pain based state space (Jazayeri and Afraz, 2017). We hypothesize that such an induced discrepancy with natural states leads to reduced efficacy and increased side-effects. One of the main potential benefits of closed-loop stimulation would be to modulate neural signals to stay within natural bounds of information processing as seen in endogenous pain-free epochs.

Finally, the proposed framework assumes that optimal biomarkers come from neural signals. However, there are many other correlates of pain that provide useful signals. For example, the RESTORE trial matches different spinal cord stimulation parameters with different patient body positions, determined from an implanted 3D accelerometer (Schultz et al., 2012). Adapting stimulation to time of day, medication timing, sleep metrics, and other external variables would also improve intervention efficacy. Broadly speaking, we acknowledge that open-loop paradigms still incorporate a form of feedback, but on the timescale of clinic visits. Ultimately, all stimulation protocols for pain including personalized closed-loop models are designed based on offline analysis by the healthcare team. The most important “biomarker” relevant for determining efficacy will always be the patients' self-report of pain.

## Author contributions

PS and TV developed the hypothesis, elaborated the theory, wrote the manuscript and created the Figures. HD provided input to the hypothesis and theory. EC provided the framework for the theory, and edited the manuscript.

### Conflict of interest statement

The authors declare that the research was conducted in the absence of any commercial or financial relationships that could be construed as a potential conflict of interest.

## Footnotes

^1^*IASP Classification of Chronic Pain, 2nd Edn. (Revised) - IASP*. Available online at: https://www.iasp-pain.org/PublicationsNews/Content.aspx?ItemNumber=1673&navItemNumber=677 (Accessed Dec 5, 2017).
